# The extent and nature of television food and non-alcoholic beverage advertising to children during chinese New Year in Beijing, China

**DOI:** 10.1186/s12889-022-13801-w

**Published:** 2022-07-26

**Authors:** Nan Lei, Zechen Liu, Lin Xiang, Lihong Ye, Juan Zhang

**Affiliations:** 1 School of Population Medicine and Public Health, Peking Union Medical College / Chinese Academy of Medical Science, 100730 Beijing, China; 2 Faculty of Medicine, Memorial University of Newfoundland, A1C 5S7 St. John’s, Canada; 3 Department of Biostatistics, Harvard T.H. Chan School of Public Health, 677 Huntington Avenue, 02115 Boston, MA USA

**Keywords:** Childhood obesity, Food advertising, Food marketing, Food promotion

## Abstract

**Background:**

Exposure to food and non-alcoholic beverage advertisements (F&B ads) on television, which can affect children’s nutrition knowledge, food consumption, diet quality, and purchasing preferences, is one aspect of the obesogenic environment. This aspect has been well-studied and assessed in many countries. In China, however, only few studies have been done in earlier years and all of them were focus on regular days. This study aimed to assess the extent and nature of F&B ads on television (TV) during the public holiday directed towards children aged 4–14 years in Beijing.

**Method:**

Top 3 channels viewed by children aged 4–14 years in Beijing were selected by TV viewership data, survey, and expert consultation. Each channel was recorded for 7 days (24 h) during the public holiday of the Chinese New Year in 2019. F&B ads were coded and analyzed following the adapted food promotion module of INFORMAS protocol. Three nutrient profile models were used to classify F&B ads as healthy or unhealthy F&B ads.

**Results:**

Of the 10,082 ads in 504-hour recorded programs, 42.9% were F&B ads. The hourly average ads and F&B ads per channel were 19.8 (SD 15.32) and 8.6 (SD 9.84), while that was higher on the national children’s channel (17.15, SD 12.25) than other channels (*p *< 0.05). Of F&B ads classified with the three nutrient profile models, more than 55% were unhealthy for children. The categories most frequently advertised were savory snacks, milk drinks, nonpermitted milk drinks, cakes/sweet biscuits, and beverages. Unhealthy F&B ads were more likely to use promotional characters, brand benefit claims, and health claims than permitted F&B ads (*p* < 0.05).

**Conclusions:**

Children in Beijing were exposed to a high proportion of unhealthy F&B ads during the Chinese New Year holiday. Our findings support the need to assess and regulate TV F&B ads marketing for children.

## Background

Overweight and obesity have become significant concerns worldwide. In China, the prevalence of overweight and obesity among children and adolescents aged 7 to 18 increased at an alarming rate from 1.5 to 19% between 1985 and 2020 [1, 2]. Excess weight during childhood and adolescence remains one of the most critical issues in China. Furthermore, overweight and obese children are likely to remain obese into adulthood and develop noncommunicable diseases [2].

Exposure to food and non-alcoholic beverage advertisements (F&B ads) on television (TV), which is one aspect of the “obesogenic” environment, may shape individuals’ food choices [3]. It can affect children’s nutrition knowledge [4], food consumption [5], diet quality [6], and purchasing preferences [7] and thus contribute to childhood obesity [8]. Researchers also reported that F&B ads used persuasive techniques to change children’s understanding of and feelings about the products, resulting in higher unhealthy food consumption [7, 9–11]. Given child-directed F&B ads disproportionately promotes products high in sugar, fat, and sodium [12], the World Health Organization (WHO) called on member states to implement comprehensive restrictions on F&B marketing to children in 2010 [13]. The WHO Regional Office for the Western Pacific Region, outlined strategies in 2014 to urge member states to regulate the marketing of unhealthy F&B and the practices of some F&B industries [14]. One study in the UK showed that 2 years following the implementation of regulation on F&B marketing to children, unhealthy F&B commercials decreased by 2.2% and healthy F&B commercials increased by 0.5% [15]. Although regulation is an effective practice to reduce F&B ads exposure, one study claimed that progress achieved by governments, industries, and other sectors varies a lot and generally is less robust than expected [16].

Although ads on other platforms (e.g., social media and steaming media) increasing quickly, TV have widely range of audience and remains its strong influence [17]. F&B marketing to children on traditional media, specifically television, has been well-studied and assessed in many countries [8, 18–26]. In China, however, only few have been done to assess the extent and nature of F&B ads for children during regular days in earlier years [27, 28], while Chinese studies have documented an increasing intake in sweetened beverages and unhealthy snack [29, 30]. It was reported that Chinese children and adolescents who paid attention to commercials were more likely to ask for snacks and buy snacks seen on TV [31]. Meanwhile, the propaganda of F&B industry together with celebrity endorsement, bluffing brand effects and nutrient claims in F&B ads has altered children’s cognitive choice and attention to F&B products [32]. Researchers discovered that after watching F&B products advertised on television, children had a strong spontaneous recall for the products advertised, particularly for the nutrient-poor, energy-dense products and children with higher information processing skills tended to recall more brands [25].

Studies in other countries suggested that children had different viewership patterns on holidays and regular days [27, 33]. As the Chinese New Year period had the highest viewership and most prolonged viewing period for all age groups [34–38], additional evidence on F&B advertising and marketing techniques used to target children and adolescents in China during holidays could help constitute a baseline to measure the extent and nature of F&B ads and to inform policy development. The International Network for Food and Obesity/non-communicable diseases Research, Monitoring and Action Support (INFORMAS) methodology has been widely used world widely. It was established to monitor, benchmark, and support the progress of national governments and private sector actions in improving healthy food environment, including regulation of unhealthy F&B marketing to children and adolescents [39].

This study applying the INFORMAS framework aimed to assess the extent and nature of F&B advertising on TV during Chinese New Year directed towards children aged 4–14 years in Beijing, which has a higher rate of childhood obesity in China. In particular, this study examined : (1) the frequency of F&B ads; (2) the type and nutritional quality of F&B advertised; and (3) the persuasive marketing techniques used by F&B ads.

## Methods

The latest protocol (updated in November 2017) of the INFORMAS food promotion module [40] was adapted to sample, record, and assess F&B ads on TV. Ads included paid commercial messages that were broadcasted before, during, or after television programs, but excluded placement and sponsor ads. The age group for children was defined as 4–14 years according to the 2014–2019 *China TV Rating Yearbook* [34–38, 41]. Peak time was defined as prime time between 7 and 11 pm [42] and subprime time between 12 and 2 pm [38]. The rest time were defined as non-peak time. INFORMAS protocol was also tailored to the Chinese contexts. For example, we used 15 types of TV program categories referring to the *China TV Rating Yearbook *[38].

### Data sampling and collection

Data were defined as TV F&B ads broadcasting during the Chinese New Year falling in February 2019 in Beijing. We recorded TV program of three top channels targeting children aged 4–14 years old. Firstly, the most popular channels watched by children were identified by viewership data from the 2014–2019 China TV Rating Yearbooks. An online survey was conducted among parents of 4–14 children in Beijing on WJX (https://www.wjx.cn/), China’s largest online survey platform, to confirm the most popular channels watched by children. We further consulted experts to ensure that the selection of top three channels watched by children are sensible and reliable. One local channel, i.e., Beijing Kaku cartoon channel, primarily targeting children, and two national channels, i.e., China Central Television 14, primarily targeting children and China Central Television 10, targeting the general public, yet popular with children, were identified.

Previous studies showed that the highest TV viewership in China appeared from the Chinese New Year’s Eve to the 5th day and on the 15th day of the first lunar month [34–38]. Therefore, 168 h per channel (3 channels, 7 days, 24 h each day) of TV programming were recorded by a third-party institution (Qingcai Media Company) for 6 continuous days from Feb 4th, the Chinese New Year’s Eve, through Feb 9th, the 5th day of the first lunar month and 1 day on Feb 19th of 2019, i.e., the 15th day of the first lunar month which was also known as the Lantern Festival. These days are generally considered to have the highest viewership throughout the year[34–38].

### Data coding

Two coders were trained and independently coded the ads. Coders were recruited from students with the Peking Union Medical College. The training included the study’s background and methodology, as well as the identification of F&B ads, three nutrient profile models, and specific marketing tactics. Before beginning the coding process, coders used sample ads to ensure that they understand the coding procedure approach. To reduce data-entry errors, data were entered by double entry. Data were entered into Microsoft Office Excel (2010 version). Intercoder reliability was 96.8% between 2-coder groups and 100% with the INFORMAS Secretariat. TV stations, companies, or other third parties played no role in extracting information from the database.

All F&B ads were coded according to the adapted INFORMAS food marketing module[40, 43, 44]. Each ad content information was coded independently. An F&B ad was included if it advertised: (1) specific F&B products; (2) F&B companies or brands; and (3) supermarkets or restaurants, which include fast-food restaurants such as KFC. Ads that do not comply with the inclusion criteria were excluded. If two or more products were shown in the same ad, the one with a more significant presence was chosen. If all products were equally presented, then the product in the central position was selected. A F&B ad was considered unhealthy if it included at least one unhealthy F&B product.

Marketing technique was considered present if at least one of the techniques was used. An ad could contain more than one specific marketing technique. All persuasive marketing tactics in each F&B ad were assessed and divided into four categories: promotional characters, brand benefit claims, health claims, and premium offers. Given the wide use of “scenes of life embedded” in the Chinese ads, we added it as one of the marketing techniques [43] in promotional characters. “Gifts and collectibles” and “loyalty programs” were excluded from the marketing technique of premium offer because both tactics were not allowed to broadcast according to the regulations of China Central TV stations [45].

Three nutrient profile models were used to classify F&B ads according to the nutrient information: the INFORMAS food system [40], the WHO Nutrient Profile Model for the Western Pacific Region [46] and the Guidelines on Snacks for Chinese Children and Adolescents (2018) [47], abbreviated as INFORMAS, WHO-WPRO and GSCCA respectively. The nutrients contents of each product were verified from the Nutrition Facts Panel on the package, or the official information accessed from the websites of the manufacture and compared against the corresponding thresholds of nutrients associated with each model. One product was classified as “unhealthy” when it did not comply with the specific nutrient criteria. Notably, GSCCA is not a dichotomy classification. It categorizes products high in fat, salt, sugar, and energy as “limited consumption”, products relatively nutrient-rich with moderate levels of salt, sugar, and energy as “appropriate consumption”, and products with rich nutrient and low levels of salt, sugar, and energy as “regular consumption”. We defined both former groups as unhealthy and “regular consumption” as healthy (referred to Table 2 for specific classification). An F&B product may be defined as healthy or unhealthy in different categories. For example, full cream milk with fat more than 4 g/100ml was classified as “regulate consumption” in the GSCCA, “permitted milk drinks” in WHO-WPRO, and “uncore full cream milks and yogurts (> 3 g fat/100 g)” in the INFORMAS food system. Therefore, full cream milk was considered unhealthy as per INFORMAS food system, while healthy as per WHO-WPRO and GSCCA.

### Data analysis

Data analyses were conducted using the statistical software SAS 9.0. The extent of F&B ads was described as the mean F&B ads per hour for each channel, the type of channels, and the type of audience across different periods (peak time vs. nonpeak time) using an independent samples t-test with a 95% confidence interval. The proportion and persuasive marketing technique of healthy and unhealthy F&B ads were compared through each nutrient profile models using Chi-squared test at a significant level of α = 0.05.

## Results

### Food and non-alcoholic beverage advertising

A total of 10,082 ads were identified throughout 508 h of TV programming, and 4376 (43.40%) were F&B ads (Table 1). The average number of ads was 19.8 (SD 15.32) per hour per channel and 8.6 (SD 9.84) for F&B ads. On average, two national channels had more F&B ads during the peak time, (*n* = 14.74 ads/h, SD 9.01) than the nonpeak time (10.50 ads/h, SD 5.13, *p* < 0.05). While there was no significant difference between peak time and nonpeak time in the local channel (peak time: *n* = 3.4 ads/h, SD 2.03; nonpeak time: *n* = 2.77 ads/h, SD 4.25). There is no statistically significant difference between the peak time (*n* = 13.11 ads/h, SD 10.65) and nonpeak time (*n* = 9.06 ads/h, SD 7.16). Notably, as all the F&B ads in the local children’s channel were assessed as unhealthy by all three models, further data analysis were only for F&B ads on the two national channels.

**Table 1:** Numbers and means of all ads and F&B ads on Beijing TV, according to the ad type and channel

	Type of ads			F&B ads/h						
				Total		Peak Time		Nonpeak Time		
	Total ads (n)	F&B ads (n)	F&B ads (%)	Mean	SD	Mean	SD	Mean	SD	***P*** value*
**Channel**										
National children’s channel	4449	2893	65.03	17.15	12.25	22.83	4.64	15.35	0.84	< 0.05
National general channel	3740	991	26.49	5.90	4.39	6.64	1.26	5.65	1.27	< 0.05
Local children’s channel	1893	492	25.99	2.93	3.43	3.40	2.03	2.77	4.25	> 0.05
Total	10,082	4376	43.40	8.7	9.84	10.96	9.17	7.92	6.04	< 0.05
**Type of channel**										
National broadcast	8189	3884	47.42	11.53	10.74	14.74	9.01	10.50	5.13	< 0.05
Local broadcast	1893	492	25.99	2.93	3.43	3.40	2.03	2.77	4.25	> 0.05
**Type of audience**										
Children	6342	3385	53.37	10.04	11.44	13.11	10.65	9.06	7.16	> 0.05
General	3740	991	26.49	5.90	4.39	6.64	1.26	5.65	1.27	< 0.05

*Ads* advertisements, *TV* television, *F&B ads* food and non-alcoholic beverage advertisements, *WHO-WPRO *WHO Regional office for the Western Pacific

### Nutrient profile models of food and non-alcoholic beverages advertised

Out of the 4376 F&B ads, 340 sponsor ads or placement ads were excluded from the data analysis (7.77%). The remaining 4036 F&B ads were classified by three nutrient profile models.

### INFORMAS food system

Out of the 4036 F&B ads, 67.24% (*n* = 2714) of F&B ads were considered uncore, with excessive total fats, saturated fat, sugars, and salt/sodium. Only 7.68% (*n* = 310) were core F&B ads, and 25.07% of F&B ads were defined as miscellaneous groups, including baby and toddler formula milk (*n* = 734, 18.19%), tea or coffee (*n* = 201, 4.98%) and recipe additions (*n* = 57, 1.41%) (Table 2).

**Table 2:** Proportion of healthy and unhealthy F&B ads and number of F&B ads in each group on Beijing TV based on the INFORMAS food system, WHO-WPRO and GSCCA

**Proportion and number of F&B ads based on the three food categories**			
**Proportion of healthy and unhealthy F&B ads based on the INFORMAS food system**			
Healthy (core F&B products)		310	7.68
Unhealthy (uncore F&B products)		2714	67.24
Miscellaneous		1012	25.07
** Proportion of each group based on the INFORMAS food system**			
** Core F&B products**			
Breads, rice and rice products without added fat, sugar or salt, noodles (exclude fried), plain starch products (e.g., starch balls), plain biscuits and crackers		5	0.12
Low-sugar and high-fiber breakfast cereals (< 20 g sugar/100 g and > 5 g dietary fiber/100 g)		27	0.67
Fruits and fruit products without added fat, sugars or salt (including fresh, tinned in natural juice, and dried), including fruit juices containing ≥ 98% fruit		83	2.06
Meat and meat alternatives – include meat, poultry, fish, legumes, tofu, eggs and raw unsalted nuts		56	1.39
Oils high in mono- or polyunsaturated fats (olive oil, sunflower oil, soybean oil, plant-based margarine and spreads), and low-fat savory sauces (< 10 g fat/100 g).		108	2.68
Bottled water (include unflavored mineral and soda waters)		31	0.77
** Uncore F&B products**			
Sweet breads, cakes, muffins, sweet buns, sweet biscuits, sweet glutinous rice balls or cakes, high-fat savory biscuits, pies and pastries, sweet sticky rice or rice pudding.		431	10.68
Savory snack foods (added salt or fat) – includes chips, dried spicy peas, fruit chips, savory crisps, extruded snacks, popcorn (exclude plain), salted or coated nuts, other fried snacks (e.g., shrimp crackers)		839	20.79
Sweet snack foods – include jelly, sugar-coated dried fruits or nuts, nut- or seed-based bars and slices, sweet rice bars, and tinned fruit in syrup		219	5.43
Full cream milks and yogurts (> 3 g fat/100 g) and cheese (> 15 g fat/100 g, and high-salt cheeses, including halloumi and feta) and their alternatives, e.g., soy		458	11.35
Chocolate and candy – including marshmallows, sugar (all types), and chewing gum (excluding sugar-free varieties)		108	2.68
Fast food (not only healthier options advertised), e.g., burgers, fries, soft drinks		3	0.07
High-fat/high-salt meals – frozen or packaged meals (> 6 g saturated fat/serving, > 900 mg sodium/serving). Also including steamed buns (excluding sweet buns), wantons and dumplings usually fried before consumption.		43	1.07
Sugar-sweetened drinks – include soft drinks, sweetened tea drinks, sports/electrolyte drinks, powdered flavor additions (e.g., sweetened tea or coffee powders)		613	15.19
** Miscellaneous F&B products**			
Recipe additions (including soup cubes, oils, dried herbs and seasonings)		57	1.41
Vitamin/mineral or other dietary supplements, and sugar-free chewing gum		20	0.50
Tea and coffee (excluding sweetened powder-based teas or coffees)		201	4.98
Baby and toddler milk formula		734	18.19
**Proportion of healthy and unhealthy F&B ads based on the WHO-WPRO Nutrient Profile Model**			
Healthy (permitted F&B products)		914	22.65
Unhealthy (nonpermitted F&B products)		2331	57.76
Excluded from classification		791	19.86
** Proportion of each group based on the WHO-WPRO Nutrient Profile Model**			
** Permitted F&B products**			
Butter and other fat and oils		108	2.68
Bread, bread products and crisp breads		146	3.62
Fresh or dried noodles, pasta, rice and grains		5	0.12
Fresh and frozen meat, poultry, fish and similar		56	1.39
Fresh and frozen fruit, vegetables and legumes		55	1.36
Milk drinks		458	11.35
Other beverages		31	0.77
Processed fruit, vegetables, and legumes		28	0.69
Ready-made and convenience foods and composite dishes		27	0.67
** Nonpermitted F&B products**			
Chocolate and sugar confections, energy bars, and sweet toppings and desserts		108	2.68
Cakes, sweet biscuits and pastries, other sweet bakery products, dry mixes for making such powers		285	7.06
Energy drinks, tea and coffee		221	5.48
Milk drinks		361	8.94
Other beverages		252	6.24
Processed meat, poultry, fish and similar		24	0.59
Savory snacks		1058	26.21
Sauces, dips, and dressings		22	0.55
** Not included**			
Formula milk (12–36 months)		734	18.19
Recipe additions		57	1.41
**Proportion of healthy and unhealthy F&B ads based on the Guidelines on Snacks for Chinese and Adolescents (2018)**			
** Consumption frequency based on the GSCCA**			
Healthy (regular consumption)		602	14.92
Unhealthy (appropriate consumption and limited consumption)		2281	56.52
Not included from classification		1153	28.57
** Proportion of each group based on the GSCCA**			
** Regular consumption**			
Meat and egg products		56	1.39
Wheat and rice products		5	0.12
Soy and bean products		145	3.59
Vegetable and fruit products		83	2.06
Milk and milk products		313	7.76
Nut and seed products		0	0
Tubers and related products		0	0
Beverages		0	0
** Appropriate consumption**			
Meat and egg products		26	0.64
Wheat and rice products		173	4.29
Soy and bean products		0	0
Vegetables and fruits		608	15.06
Milk and milk products		0	0
Nut and seed products		0	0
Tubers and related products		0	0
Beverages		573	14.20
Candy and ice cream		0	0
** Limited consumption**		0	0
Meat and egg products		24	0.59
Wheat and rice products		490	12.14
Vegetable and fruit products		0	0
Milk and milk products		0	0
Nut and seed products		0	0
Tubers and related products		0	0
Beverages		60	1.49
Candy and ice cream		327	8.10
** Not included**			
Oil		108	2.7
Formula milk		734	18.19
Bottled water		31	0.77
Tea		201	4.98
Recipe additions		79	1.96

*Ads* advertisements, *TV *television, *F&B ads *food and non-alcoholic beverage advertisements; national children’s channel, China Central Television Channel 14; national general channel: China Central Television Channel 10; local children’s channel: Beijing Kaku cartoon channel; peak time: 12-2pm and 7-11pm; nonpeak time: midnight-12pm, 2-7pm, 11pm-midnight

*Comparing peak time with nonpeak time, independent samples t-test. *P *< 0.05 is significant

The F&B categories most frequently advertised are savory snacks (e.g., chips, flavored seaweed, shrimp crackers; *n* = 839, 20.79%), sugar-sweetened drinks (e.g., sweetened tea powders, soft drinks; *n* = 613, 15.19%), full-cream milk and their alternatives (e.g., whole fat milk, soy; *n* = 458, 11.35%), sweetbreads, cakes and biscuits (*n* = 431, 10.68%), and sweet snacks (e.g., sweet jelly; *n* = 219, 5.43%). All the five F&B groups were defined as uncore products.

**Fig. 1:**
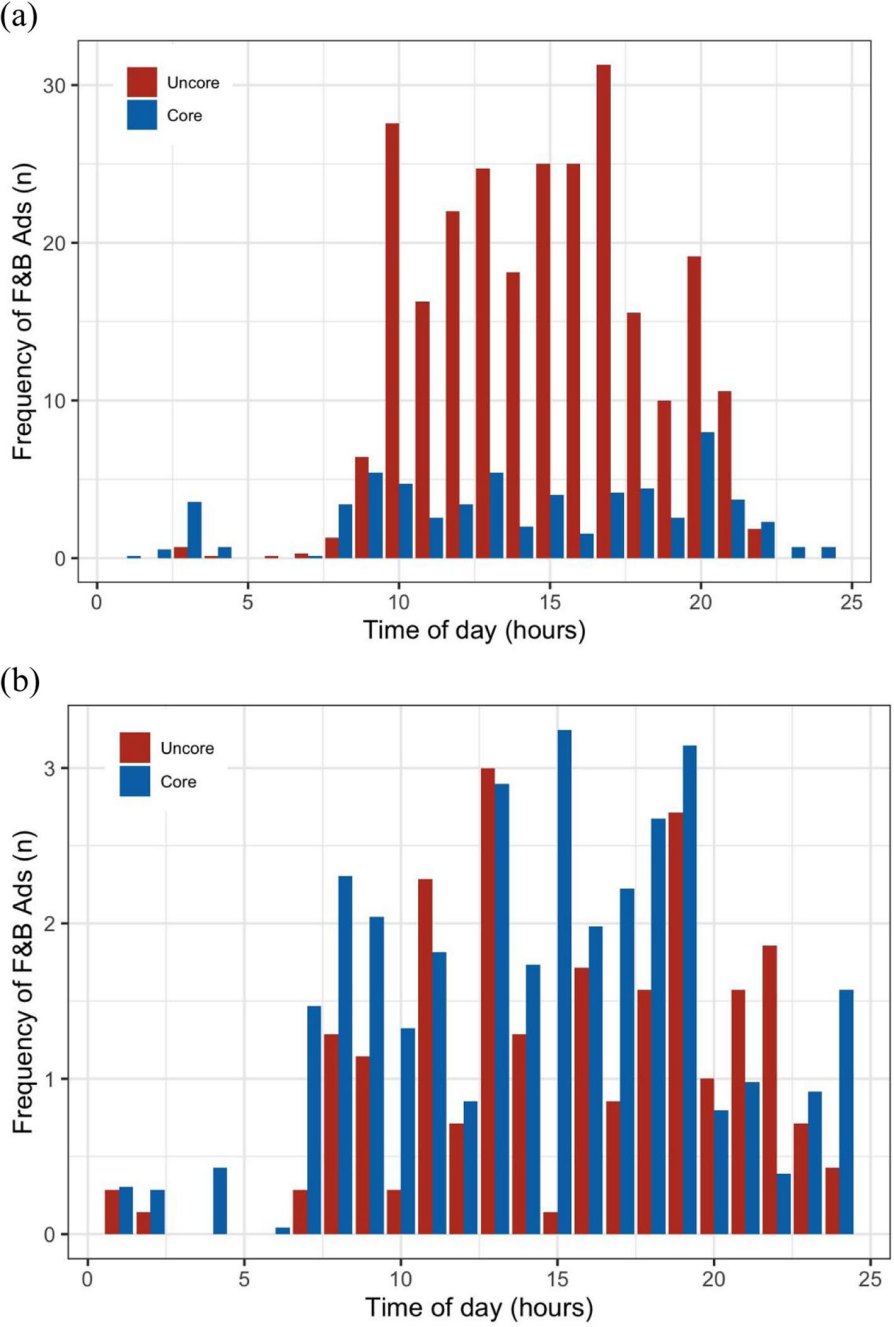
Comparison of the hourly number of F&B ads on TV as per INFORMAS food system: (**a**) national children’s channel and (**b**) national general channel. Ads: advertisements; TV: television; F&B ads: food and non-alcoholic beverage advertisements; national children’s channel: China Central Television Channel 14; national general channel: China Central Television Channel 10

As shown in Fig. 1 (a) and (b), the hourly frequency of F&B ads considered uncore was higher than those considered core in the national children’s channel (*p* < 0.05). For the national general channel, the hourly frequency of core F&B ads was higher than the uncore F&B ads, while there was no significant difference (*p* > 0.05).

### WHO-WPRO Nutrient Profile Model

Using the WHO-WPRO Nutrient Profile Model, out of the 4036 F&B ads, 57.76% (*n* = 2331) of products were considered nonpermitted due to excessive content of total fat, saturated fat, total sugar, artificial sweeteners, salt, and/or energy, while 22.65% (*n* = 914) were considered permitted. Formula milk (*n* = 734, 18.19%) and recipe additions (*n* = 57, 1.41%) were not considered either permitted or nonpermitted.

The most frequently advertised F&B products were nonpermitted savory snacks (e.g., chips, processed seaweed, crisps; n = 1058, 26.21%), permitted milk drinks (e.g., whole fat milk, soy, *n* = 458, 11.35%), nonpermitted milk drinks (e.g., sweet milk, flavored almond milk; *n* = 361, 8.94%), cakes, sweet biscuits and pastries (*n* = 285, 7.06%) and nonpermitted other beverages (e.g., tea, coffee, energy drinks; *n* = 252, 6.24%).

**Fig. 2:**
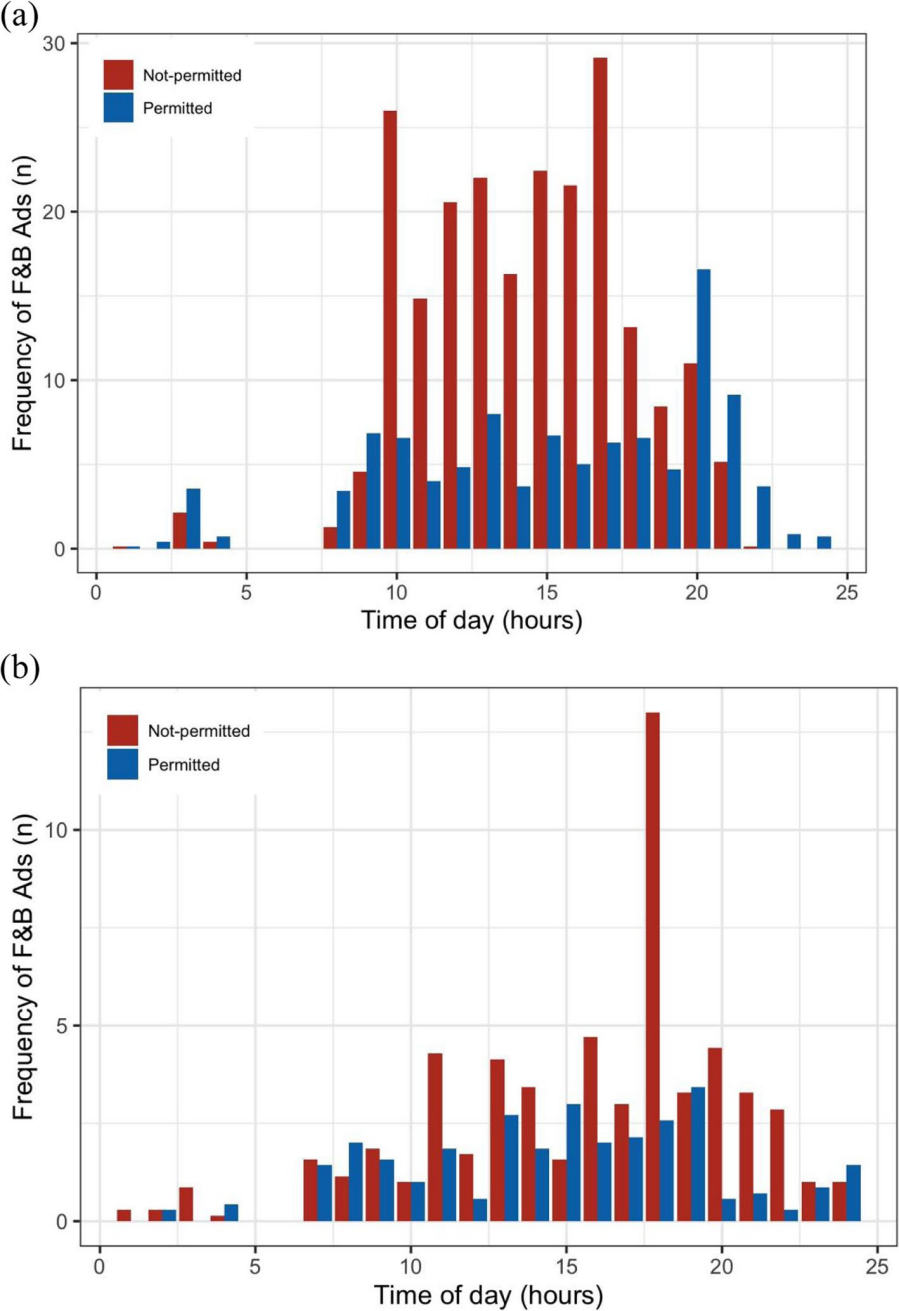
Comparison of the hourly number of F&B ads on TV as per WHO-WPRO: (**a**) national children’s channel and (**b**) national general channel. Ads: advertisements; TV: television; F&B ads: food and non-alcoholic beverage advertisements; national children’s channel: China Central Television Channel 14; national general channel: China Central Television Channel 10

As shown in Fig. 2 (a) and (b), the hourly frequency of F&B ads considered not permitted was higher than that for F&B ads considered permitted on the national children’s channel (*p* < 0.05). For the national general channel, the hourly frequency of nonpermitted F&B ads was also higher than permitted F&B ads (*p*< 0.05).

### GSCCA

Out of the 4036 F&B ads, 57.76% (*n* = 2331) were classified as “limited consumption or appropriate consumption (unhealthy)”, while 22.65% (*n* = 914) were classified as “regular consumption(healthy)” by GSCCA. Some F&B ads that were not included in the model, such as formula milk (*n* = 734, 18.19%), tea (*n* = 201, 4.98%), bottled water (*n* = 31, 0.77%), oil (*n* = 108, 2.7%) and recipe additions (*n* = 57, 1.41%).

The F&B groups most frequently advertised were “appropriate consumption of vegetable and fruit products” (e.g., processed seaweed; *n* = 608, 15.06%), “appropriate consumption of beverages (e.g., sweet almond milk, flavored yogurt; *n* = 573, 14.20%)”, “limited consumption of wheat and rice products (e.g., shrimp crackers; *n* = 490, 12.14%)”, “limited consumption of candy and ice cream (*n* = 327, 8.10%)”, and “regular consumption of milk and milk products (e.g., whole fat milk; *n* = 313, 7.76%)”. Only “regular consumption of milk and milk products” was considered healthy.

**Fig. 3:**
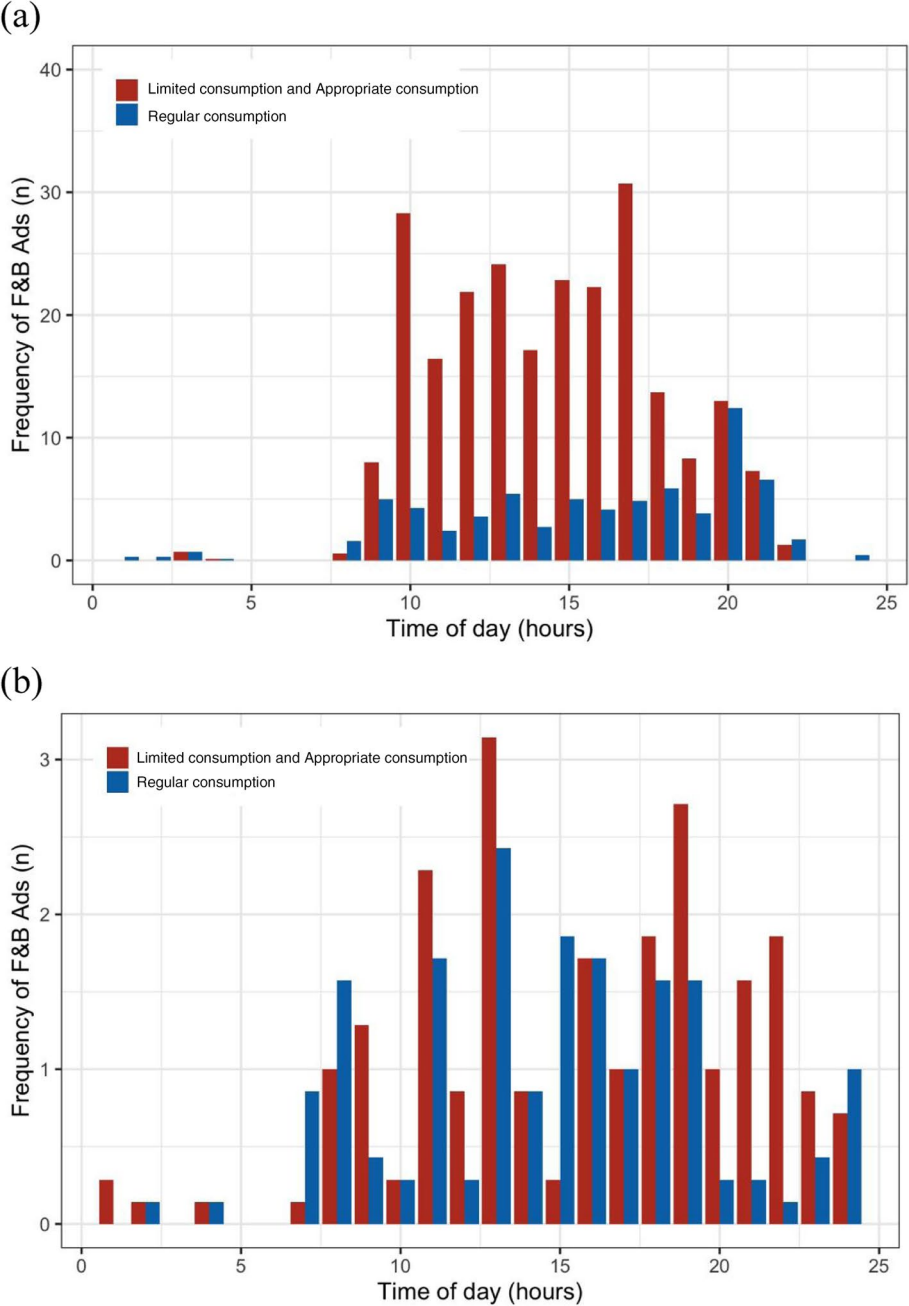
Comparison of the hourly number of F&B ads on TV as per the Guidelines on Snacks for Chinese Children and Adolescents (2018): (**a**) national children’s channel, and (**b**) national general channel. Ads: advertisements; TV: television; F&B ads: food and non-alcoholic beverage advertisements; national children’s channel: China Central Television Channel 14; national general channel: China Central Television Channel 10

As shown in Fig. 3 (a) and (b), the hourly frequency of F&B ads for products considered unhealthy was higher than that considered healthy in the national children’s channel (*p *< 0.05). It holds true for the national general channel, (*p *< 0.05).

### Persuasive techniques for the marketing of food and non-alcoholic beverage advertisements

**Fig. 4:**
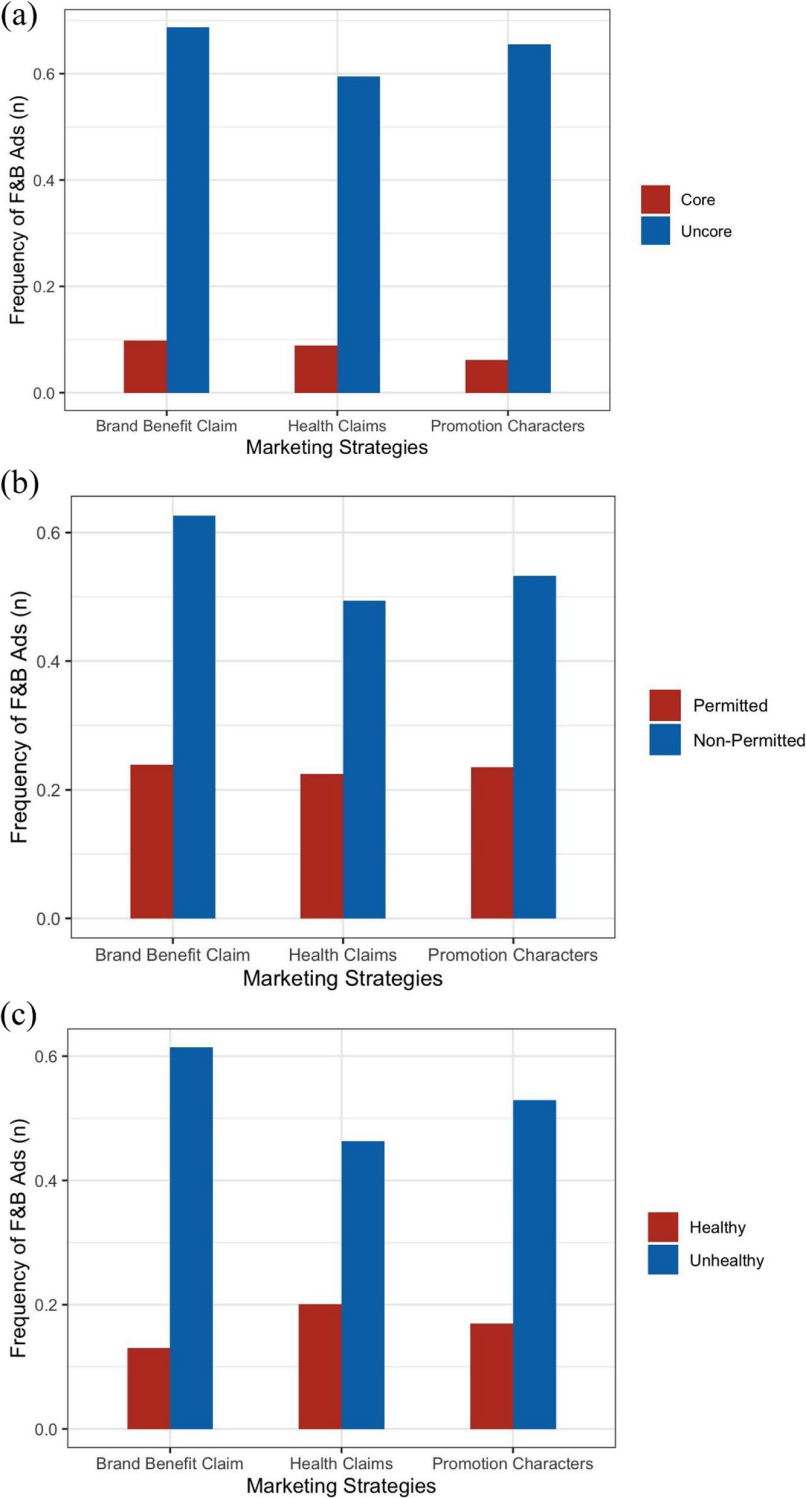
The proportion of F&B ads (*n* = 4036) on TV to which children aged 4–14 years are likely to be exposed. Each type of persuasive marketing technique is displayed as per (**a**) the INFORMAS food system, (**b**) WHO-WPRO, and (**c**) GSCCA. An ad could have more than one technique used; *p *< 0.05 (χ2 test for the differences between healthy and unhealthy groups within each marketing technique). Ads: advertisements; TV: television; F&B ads: food and non-alcoholic beverage advertisements

As displayed in Table 3, all F&B ads used a brand benefit claim and promotional characters when promoting the products (n = 4036). As many as 25.15% of F&B ads used health claims, including nutrient claims, nutrition function claims and other claims. Figure 4 shows that F&B ads considered unhealthy were more likely to include promotional characters, brand benefit claims, and health claims in their marketing regardless of the nutrient profile model (*p* < 0.05). Specifically, promotional characters, benefit claims and health claims were present in 65.54%, 68.75%, and 59.68% of F&B ads considered uncore as per INFORMAS, versus 6.18%, 9,85% and 8.85% of F&B ads considered core; They were in 53.34%, 62.63% and 49.35% of nonpermitted ads as per WHO-WPRO versus 23.47%, 23.91% and 22.46% of permitted ads; They were in 52.85%, 61.39% and 46.31% of unhealthy ads as per GSCCA versus 16.96%, 12.96% and 20.08% of healthy F&B. None of the “premium offers” techniques were presented in recorded F&B ads.

**Table 3:** Proportion of F&B ads on Beijing TV, according to the marketing technique used and type of model^a^

			INFORMAS food system			WHO-WPRO Nutrient Profile Model					GSCCA		
**Marketing technique**	**n**	**%**	**Core**	**Uncore**		**Permitted**		**Nonpermitted**			**Healthy**		**Unhealthy**
**Promotional characters**	**No. of F&B ads** **(n 4036, 100%)**												
Use cartoon/company-owned characters, famous characters, licensed characters, or celebrities	2011	49.83	27	1421		631		837			458		1010
Use scenes of major historical rituals or events, festivals, etc.	1335	33.08	33	872		33		1025			33		872
History honors, awards, and achievements, etc. that the product or the company achieved	359	8.89	166	21		166		192			84		21
Scenes of life embedded	2815	69.75	241	2192		700		1792			440		1904
Recommended for target groups (e.g., “for infants” or “for kids”)	690	17.10	0	0		0		0			0		0
Joint promotion with other brands or products	699	17.32	22	677		326		373			326		373
**Brand benefit claims**	**No. of F&B ads** **(n 4036, 100%)**												
Sensory characteristics (taste, texture, appearance, aroma, etc.)	1635	40.51	153	1246		308		1283			86		1237
New product development	3	0.07	0	3		0		3			0		3
Describe product details, such as “produced using pure milk”	1768	43.81	228	657		532		545			392		380
Emotional appeal (fun, happiness, popularity, such as “Everyone likes XX”)	1814	44.95	27	1767		327		1487			154		1660
Exaggerating (claiming to be better than other products)	242	6.00	130	82		139		103			76		73
Convenient	0	0.00%	0	0		0		0			0		0
**Health claims**	**No. of F&B ads** **(n 1015, 25.15%)**				^a^								
	1010	25.02	100	569		100		607			72		569
Nutrition claims (e.g., low fat)	817	20.24	61	636		61		686			33		656
Claims of higher or lower nutrient content (e.g., reduced fat)	60	1.49	34	171		179		26			179		26
General health claims (e.g., a healthy diet)	678	16.80	74	473		83		494			60		464
Nutrition and other functional claims (e.g., calcium is good for bones)	1015	25.15	56	671		360		405			332		367
Claims to reduce disease risk	255	6.32	0	0		0		0			0		0
Other claims (e.g., organic)	661	16.38	73	154		227		1			227		0
**Premium and price-related offers**													
	0	0.00	0	0		0		0			0		0

*Ads* advertisements, *TV *television, *F&B ads *food and non-alcoholic beverage advertisements

^a^ Advertisements could employ one or more marketing techniques

## Discussion

This present study indicated that children in Beijing were exposed to a large number of unhealthy F&B ads on television during the Chinese New Year. Over half of F&B advertised were not healthy according to three different nutrient profiling models. Unhealthy F&B ads used more persuasive marketing techniques than healthy F&B ads. The findings suggest F&B products advertised on television targeted to children in Beijing are well-persuasive and mainly have poor nutrient profiles, which may exacerbate the epidemic of childhood obesity.

During the Chinese New Year, children in Beijing were exposed to approximately 20 ads per hour and 42.9% were F&B ads. The results are much higher than that reported in other countries, for example, Argentina (14.6 ads per hour, with 16.96% F&B ads), New Zealand (9.1 ads per hour, with 17.3% F&B ads), Brazil (7.5 ads per hour, with 18.1% F&B ads), Mexico (4.24 ads per hour, with 20.7% F&B ads) and Costa Rica (3.7 ads per hour, with 20.7% F&B ads) [19–24, 27, 48, 49], but comparable with that reported in previous Chinese studies in earlier years [8, 28, 50], for example, Xi’an (28.8 ads per hour, with 26.43% F&B ads), Heilongjiang (28.5 ads per hour, with 18.59% F&B ads), Shanghai (31.4 ads per hour, with 26.43% F&B ads) and mainland average (23.7 ads per hour, with 32.3% F&B ads) [8, 28, 50].

Unhealthy F&B products accounted for more than half of F&B ads as per three different nutrient profile model, i.e., INFOMAS, WHO-WPRO, and GSCCA. The most frequently advertised food and non-alcoholic beverages were savory snacks and sweet biscuits, followed by formula milk, sweet beverages, and dairy products, while previous studies in other countries and in some cities of China suggested beverage, chocolate, and sugar confections were the most frequently advertised product on TV [19]. Possible explanations for the differences could be: while television remains the major source of F&B marketing to children, marketing appeals to children becomes present across multiple media including broadcasting television, internet, and social media [51, 52]; recent research shows television-based F&B marketing directed at children differ between regular days and holiday seasons [25, 27]. It is worth noting that we found all F&B ads in local children channel were unhealthy products, most F&B ads in national children channel were unhealthy and a high proportion of unhealthy F&B ads in national general channel yet popular among children. The present study suggested that television-based F&B marketing directed at children predominantly promotes unhealthy foods.

The findings of the present study indicated F&B ads were accompanied by highly persuasive marketing strategies. Unhealthy F&B ads used more promotional marketing techniques than healthy ads, emphasized their attachment with children by using promotional characters and brand claims such as “scenes of life embedded” (e.g., children eat snacks in a lovely room with happy family members) and “emotional appeal” (e.g., slogans like “happy jelly”). Previous studies in other countries and some cities of China reported that premium and price-related offers were the most common marketing techniques in F&B ads [8, 28, 50] while we did not find any in the present study. A possible explanation is that national TV station regulation requires that all ads should double check if the contents include any premium and price-related offers. A quarter of F&B ads used health claims category and of which the most popular tactics are nutrition claims (e.g., high protein with low fat) and ingredients claims (e.g., DHA is good for children’s health). These marketing techniques tends to mislead children by demonstrate some attributes with the F&B products that they might not have [53]. Children are generally unaware of the persuasive goal of food marketing and mistakenly believe advertisements to be genuine and truthful, making them an easy target [11]. Existing evidence suggests children are particularly vulnerable to the effects of acute exposure to food advertising [53, 54], urgent actions should be taken to address children’s exposure to unhealthy food advertising in China.

The percentage of Chinese children who are overweight or obese nearly tripled between 2014 and 2020 [2], which should motivate towards implementing public health actions accordingly. The aetiology of obesity in children is complex and multifactorial, and therefore initiatives should address not only the individual, but also the community and the broader environment where children live in. Restricting F&B advertising is a key factor in the overweight and obesity prevention framework to reduce children’s obesity [14, 15], especially when there is evidence showing the impact of advertisement restrictions on the nutritional status of children. For example, South Korea implemented a set of regulations in 2010 to restrict unhealthy F&B marketing to children on TV. A set of thresholds were set for energy, sodium, saturated fats and sugar and unhealthy F&B ads cannot be broadcasted during children’s programming and before, during, and after programs aired from 5:00 pm to 7:00 pm [55]. In Singapore, advertising guidelines require that all F&B products promoted in marketing communications for children aged 12 and below must meet the critical nutrients for the different F&B product categories. Products that cannot be advertised to children include sugar and sugar-based products such as chocolate, as well as carbonated and non-carbonated soft drinks [56]. In China, the Advertising Law of the People’s Republic of China (2021 Amendment) includes some relevant broad requirements to protect children [57]. The newly updated Law of the People’s Republic of China on the Protection of Minors (2020 Revision) reconfirmed the significance [58]. However, in China, no specific regulations govern commercial F&B marketing targeted to children. It is urgent to advocate for regulating food and beverage marketing to children. Additional evidence is needed for specifics of the regulation in China. Nutrient Profiling Model is fundamental to define the healthfulness of F&B. The present study applied three different Nutrient Profiling Model, i.e., WHO-WPRO Nutrient Profile Model and INFORMAS food system, GSCCA. As high as 28.57% of F&B were not applicable for the GSCCA. Further efforts are needed to develop a Nutrient Profiling Model tailoring to the Chinese context.

### Strengths & limitations

To the best of our knowledge, this is the first study to examine the frequency, nutrition quality, and persuasive marketing techniques of F&B ads in Beijing during the Chinese New Year. Our study recorded 24 h of TV advertisements of three channels. By including one channel directed to both children and adults, we were able to monitor F&B marketing not only in child-directed channels, but also general public channels popularly viewed by children. Furthermore, by recording 24 h, it enabled us to monitor F&B ads throughout the day besides children’s peak viewing time slots.

Our study had several limitations. First, the selected channels may not represent channels that children watched most, however we used multiple ways to ensure the accuracy by referring to television viewership data, conducting survey with parents of children aged 4 to 14, and consulting with stakeholders from the broadcasting bureaus. Second, this study may underestimate F&B advertisements targeting children with the focus on advertisement on television only, while television remains the major channel of F&B marketing. For example, 99% of the China population above 5 years of age reports watching TV daily [38]. Third, a study had shown that children’s peak viewing time might differ from that of adults [24], the definition of prime time in China has remained the same for almost 40 years though. Lastly, the study may underestimate the nature and exposure of F&B beverage advertisement on television with the exclusion of placement and sponsor ads in the content analysis. We were aware that the duration of placement ads within TV programs varied from 3 to 448 s and all ads longer than 60 s were placement ads, which suggested that their influence could be underestimated.

## Conclusions

Our study was the first to measure extent and nature of television food and non-alcoholic beverage advertising for children aged 4–14 years in Beijing during Chinese New Year and the findings suggest it is concerning that children living in Beijing are exposed to a large number of unhealthy F&B ads according to three different nutrition profiling models and unhealthy F&B ads actively uses persuasive techniques likely to appeal to children. More stringent regulation is needed to protect children in China from the far-reaching health effects of food and beverage advertising.

## Data Availability

The datasets generated and/or analyzed during the current study are not publicly available due to the institution request which funded this study but are available from the corresponding author on reasonable request.

## Abbreviations

F&B ads: food and non-alcoholic beverage advertisements
TV: television
INFORMAS: the International Network for Food and Obesity/non-communicable diseases Research, Monitoring and Action Support
